# Complete Genome Sequence of *Mycobacterium* sp. Strain 4858

**DOI:** 10.1128/genomeA.01604-17

**Published:** 2018-02-15

**Authors:** Amar Bouam, Anthony Levasseur, Maryline Bonnet, Laurence Borand, Charles Van Goethem, Michel Drancourt, Sylvain Godreuil

**Affiliations:** a IHU Méditerranée Infection, MEPHI Aix Marseille Université IRD AP-HM, Marseille, France; b UMI233-TransVIHMI/INSERM U1175, Institut de Recherche pour le Développement (IRD) and University of Montpellier, Montpellier, France; c Epidemiology and Public Health, Institut Pasteur du Cambodge, Phom Penh, Cambodia; d Département Biopathologie Cellulaire et Tissulaire des Tumeurs, Centre Hospitalier Universitaire (CHU) de Montpellier, Montpellier, France; e Centre Hospitalier Universitaire (CHU) de Montpellier, Laboratoire de Bactériologie, MIVEGEC, UMR IRD 224-CNRS 5290, Université de Montpellier, Montpellier, France

## Abstract

*Mycobacterium* sp. strain 4858 is a nontuberculous mycobacterium isolated from sputum in a Cambodian patient with a pulmonary infection. We report the first complete 5.6-Mbp-long genome sequence of *Mycobacterium* strain 4858, with 68.24% GC content, carrying 5,255 protein-coding genes, 47 tRNAs, and 3 rRNA genes.

## GENOME ANNOUNCEMENT

*Mycobacterium* sp. strain 4858 is a pigmented slowly growing acid-fast bacillus isolated from a respiratory tract specimen collected from a patient with a pulmonary infection in Cambodia. In order to gain insight into the taxonomic position of this previously undescribed species, we analyzed its whole-genome sequence.

Strain 4858 was cultured on Middlebrook 7H11 agar supplemented with 10% (vol/vol) oleic acid-albumin-dextrose-catalase (Becton, Dickinson, Sparks, MD). The total DNA of strain 4858 was extracted on the EZ1 biorobot (Qiagen) with an EZ1 DNA tissue kit with a 50-µl elution volume. The concentration of extracted DNA measured using the Qubit assay with a high sensitivity kit (Life technologies, Carlsbad, CA, USA) was 55.5 ng/µl. Total DNA was then sequenced using MiSeq technology (Illumina, Inc., San Diego, CA) using the paired-end technique coupled with the mate pair technique. The index representation for strain 4858 was determined to be 5.42%. A total of 683,373 paired-end reads, filtered per the read qualities, were assembled using the SPAdes software (1). The resulting contigs were combined by use of SSPACE (2) assisted by manual finishing and GapFiller (3). This yielded a 5,614,132-bp draft genome with a 68.24% GC content, composed of 8 scaffolds and 12 contigs. Open reading frames (ORFs) were predicted using Prodigal (4) with default parameters. Functional annotation was achieved using BLASTp against the GenBank database (E value, 0.001; coverage, 0.7; identity, 30%) (5) and the Clusters of Orthologous Groups (COG) database (6). When no search results were found, a second round was done against the nonredundant protein sequence (nr) database using BLASTP with an E value of 1 × 10^−03^, coverage of 0.7×, and 30% identity. Noncoding genes and miscellaneous features were predicted using RNAmmer (7), ARAGORN (8), Rfam (9), Pfam (10), and Infernal (11). Of the 5,305 predicted genes, 5,255 were protein-coding genes and 5 were encoded RNAs, including 1 5S rRNA, 1 16S rRNA, 1 23S rRNA, and 47 tRNAs. A total of 4,175 genes (79.45%) were assigned putative functions (by COG or nr BLAST search), 79 genes were identified as ORFans (ORFs with no detected homology to other ORFs in the database) (1.5%), and 831 genes (15.81%) were annotated as hypothetical proteins. The genome of strain 4858 was further analyzed by *in silico* DNA-DNA hybridization (DDH) (12), with genomes exhibiting the closest 16S rRNA gene sequence similarity. The DDH values were estimated using the GGDC (Genome-to-Genome Distance Calculator) version 2.0 online tool (13). This analysis yielded a DDH value of 45% with *Mycobacterium europaeum* CSUR 1344 (GenBank accession number CTEC00000000), 34.50% with *Mycobacterium parascrofulaceum* ATCC BAA-614 (ADNV00000000), 25.60% with *Mycobacterium palustre* DSM 44572 (LQPJ01000000), 23.10% with *Mycobacterium sherrisii* BC1_M4 (MIHC00000000), and *Mycobacterium simiae* ATCC 25275 (CBMJ000000000) and 22.20% with *Mycobacterium kubicae* CIP 106428 (LQPD00000000). These data indicate that *Mycobacterium* sp. strain 4858 is related to the *Mycobacterium simiae* complex of mycobacteria, expanding this large complex to its twentieth species.

### Accession number(s).

The *Mycobacterium* sp. strain 4858 genome sequence has been deposited at EMBL under the accession number OESL01000000.
